# Prevention of violent revictimization in depressed patients with an add-on internet-based emotion regulation training (iERT): study protocol for a multicenter randomized controlled trial

**DOI:** 10.1186/s12888-018-1612-3

**Published:** 2018-02-02

**Authors:** Carolien Christ, Marleen M. de Waal, Digna J. F. van Schaik, Martijn J. Kikkert, Matthijs Blankers, Claudi L. H. Bockting, Aartjan T. F. Beekman, Jack J. M. Dekker

**Affiliations:** 1 Department of Psychiatry, VU University Medical Center/ GGZ inGeest, P.O. Box 7057, 1007 MB Amsterdam, The Netherlands; 2 Department of Research, Arkin Mental Health Care, Klaprozenweg 111, 1033 NN Amsterdam, The Netherlands; 3 Amsterdam Public Health research institute, VU University Medical Center, Van der Boechorststraat 7, 1081 BT Amsterdam, The Netherlands; 4 Academic Medical Center, Department of Psychiatry, Amsterdam Institute for Addiction Research, University of Amsterdam, Meibergdreef 9, 1105 AZ Amsterdam, The Netherlands; 5 Trimbos Institute – Netherlands Institute of Mental Health and Addiction, Da Costakade 45, 3521 VS Utrecht, The Netherlands; 6 Department of Clinical Psychology, Faculty of Social and Behavioural Sciences, University Utrecht, Heidelberglaan 1, 3584 CS Utrecht, The Netherlands; 7 Department of Clinical Psychology, Neuro- and Developmental Psychology, Faculty of Behavioural and Movement Sciences, Vrije Universiteit Amsterdam, Van der Boechorststraat 1, BT 1081 Amsterdam, The Netherlands

**Keywords:** Victimization, Depression, Emotion regulation, iERT-training, E-mental health, Internet-based intervention, Violence prevention, Multicenter randomized controlled trial

## Abstract

**Background:**

Psychiatric patients are at high risk of becoming victim of a violent crime compared to the general population. Although most research has focused on patients with severe mental illness, depressed patients have been demonstrated to be prone to victimization as well. Victimization is associated with more severe symptomatology, decreased quality of life, and high risk of revictimization. Hence, there is a strong need for interventions that focus on preventing violent revictimization. Since emotion dysregulation is associated with both victimization and depression, we developed an internet-based Emotion Regulation Training (iERT) to reduce revictimization in depressed patients. This study aims to evaluate the clinical and cost-effectiveness of iERT added to Treatment As Usual (TAU) in reducing incidents of violent revictimization among depressed patients with a recent history of victimization. Furthermore, this study aims to examine secondary clinical outcomes, and moderators and mediators that may be associated with treatment outcomes.

**Methods:**

In a multicenter randomized controlled trial with parallel group design, patients with a major depressive disorder and a history of violent victimization over the past three years (*N* = 200) will be allocated to either TAU + iERT (*N* = 100) or TAU only (*N* = 100), based on computer-generated stratified block randomization. Assessments will take place at baseline, 8 weeks, 14 weeks, and 6 months after start of treatment, and 12, 24, and 36 months after baseline. The primary outcome measure is the total number of violent victimization incidents at 12 months after baseline, measured with the Safety Monitor: an adequate self-report questionnaire that assesses victimization over the preceding 12 months. Secondary outcome measures and mediators include emotion dysregulation and depressive symptomatology. An economic evaluation with the societal perspective will be performed alongside the trial.

**Discussion:**

This study is the first to examine the effectiveness of an intervention aimed at reducing violent revictimization in depressed patients. If effective, iERT can be implemented in mental health care, and contribute to the well-being of depressed patients. Furthermore, the results will provide insight into underlying mechanisms of revictimization.

**Trial registration:**

The study is registered at the Netherlands Trial Register (NTR5822). Date of registration: 4 April 2016.

## Background

Psychiatric patients are at high risk of becoming victim of a violent crime [1, 2]. Prevalence rates of violent victimization – commonly defined as physical assault, sexual assault or threat of violence – in psychiatric patients worldwide have been demonstrated to be up to 11 times higher in comparison with the general population, with most studies reporting 3 to 6-fold elevated odds [1–3]. Recently, a Dutch prevalence study conducted by Kamperman et al. (2014) [3] showed that 19.1% of outpatients with severe mental illness (SMI) had become victim of a violent crime over the past year, as compared to 6.1% of the general population. Whilst research has focused mainly on psychiatric patients as perpetrators (e.g., [4]), several studies have demonstrated that patients are more likely to be victim rather than perpetrator of a crime [5, 6].

Victimization is a highly stressful event that can aggravate existing symptoms [7] and substantially decrease quality of life [8] in psychiatric patients. Victimization is associated with physical injury, hospitalization [7, 8], treatment resistance [9], depression, posttraumatic stress disorder, and substance abuse [7]. Furthermore, initial victimization heightens the risk of future revictimization [10, 11], and therefore might induce a vicious cycle of stressful experiences and worsening symptoms. Due to increased service use, health care use, and productivity losses, victimization has a substantial economic impact as well [12].

To date, most studies have addressed victimization in patients with severe mental illness (SMI) [6, 13, 14] and patients with substance use disorders [15, 16]. Studies that specifically address victimization in other psychiatric populations, such as outpatients with affective disorders, are scarce. The only clinical study that focused on patients with depression revealed that depressed outpatients were 3.4 times more likely to be victim of a violent crime than members of the general population [17]. In a general population study of women, a current depression predicted subsequent physical victimization, but not subsequent sexual victimization [18]. Furthermore, a meta-analysis demonstrated depressed subjects to be vulnerable to domestic violence [19].

Despite the elevated prevalence rates and harmful consequences of victimization in psychiatric patients, evidence-based interventions specifically aimed at reducing victimization risk are not available. Currently, two interventions that aim to prevent victimization in patients with dual diagnosis [20] and psychotic disorders [21] are being examined in randomized controlled trials. There remains a strong need for evidence-based interventions that focus on reducing violent victimization in other high-risk psychiatric populations [3, 6, 13, 22], including depressed patients.

To be able to develop an intervention that specifically reduces victimization in depressed patients, knowledge regarding risk factors for victimization is necessary. Unfortunately, there remains a paucity of controlled, prospective studies that systematically examine risk factors, and no risk factors of victimization in depressed patients have yet been identified. Nevertheless, several – mainly cross-sectional – studies have identified various factors that have consistently been associated with victimization and revictimization in patients with SMI: symptom severity [2, 23], alcohol and drug abuse [2, 23, 24], a history of child abuse [23], and previous victimization [11]. In the general population, a history of child abuse [25], previous victimization [10], alcohol and drug abuse [26], and dysfunctional emotion regulation [27] have been associated with victimization risk.

Dysfunctional emotion regulation is considered to be both a consequence of prior victimization and a predictor of future revictimization [27, 28]. Emotion regulation refers to “the processes responsible for monitoring, evaluating, and modifying emotional reactions, especially their intensive and temporal features, to accomplish one’s goals” [29]. Using a prospective design, Messman-Moore, Ward and Zerubavel (2013) [27] demonstrated emotion dysregulation to significantly predict subsequent revictimization in previously victimized female students. Several authors hypothesize that dysfunctional emotion regulation interferes with the ability to appraise risk situations [30–32], and may therefore impede self-protection and escape responses. Comparably, Marx et al. (2005) [28] suggested that victims of child sexual abuse adapt certain emotion regulation strategies – especially passive, avoidant behaviors – to cope with their increased level of fear and arousal. However, instead of reducing their psychological distress, the authors hypothesized that these strategies (1) reduce the effort that can be given to self-protection in dangerous situations, (2) impair risk assessment and threat detection, and (3) signal vulnerability to possible perpetrators, thereby increasing proneness to future victimization [28]. Hence, dysfunctional emotion regulation may be an important target for preventing revictimization.

Over the past decades, emotion dysregulation has been pointed out repeatedly as a perpetuating factor of depression. Difficulties in regulating negative emotions are associated with depressive symptoms in both cross-sectional and longitudinal studies [33–36]. Correspondingly, evidence of experimental studies suggests that a current or past depression is likely to coincide with emotion dysregulation [37–40]. Changes in emotion regulation have been demonstrated to partially mediate treatment outcome in treatment for several disorders [41–43], including depression: Radkovsky et al. (2014) [35] demonstrated that the successful application of functional emotion regulation skills was associated with a reduction of depressive symptoms.

In conclusion, dysfunctional emotion regulation seems to be a promising target of intervention for both victimization and depression. Therefore, we developed an internet-based emotion regulation training (iERT) that will be added to Treatment As Usual (TAU). iERT is based on the Affect Regulation Training (ART) [44]: an intensive, structured group skills training that was demonstrated to be an effective addition to Cognitive Behavioral Therapy in decreasing depressive symptoms and enhancing various emotion regulation skills in depressed patients [45]. To provide an accessible and more feasible training that can be added to TAU, we developed a guided online version of ART that consists of 6 sessions. Numerous studies have demonstrated internet-based interventions to be an effective treatment for depression [46, 47]; moreover, interventions with online guided support have been found to be equally effective as face-to-face treatment [48, 49].

iERT is a transdiagnostic online add-on training that aims to enhance emotion regulation in patients by teaching them four emotion regulation skills: (1) non-judgmental awareness of emotions, (2) acceptance and tolerance of emotions, (3) analyzing emotions, and (4) modifying emotions. By enhancing emotion regulation skills, iERT aims to reduce violent victimization. Since both depressed patients and previously victimized patients are more likely to experience dysfunctional emotion regulation and have an increased revictimization risk compared to others, we will investigate the effectiveness of iERT in a high-risk population of previously victimized, depressed patients, who are specifically likely to benefit.

### Research aims

The main purpose of this study is to evaluate the clinical effectiveness of the addition of iERT to Treatment As Usual (TAU) in reducing incidents of revictimization among patients with a major depressive disorder and a recent history of victimization. Since previous studies underline the hypothesis that ER represents an underlying mechanism leading to revictimization, we expect TAU + iERT to significantly decrease incidents of revictimization as compared to TAU alone, by enhancing ER skills.

In addition, we aim to examine the cost-effectiveness of the addition of iERT to TAU in reducing revictimization, and its effectiveness in reducing depressive symptoms and other secondary clinical outcomes. We expect the addition of iERT to significantly reduce depressive symptoms and to improve other secondary outcomes. Finally, we aim to enhance knowledge regarding the underlying mechanisms of victimization. Using a randomized, prospective, and longitudinal design, we will therefore examine the relation between victimization and changes in a variety of relevant secondary outcomes and potential mediators and moderators.

## Methods

### Design

We will conduct a multicenter two-arm randomized controlled trial (RCT) with a parallel group design, in which 200 participants will be allocated to either TAU (*N* = 100) or TAU + iERT (*N* = 100) after the first baseline assessment. The effectiveness of the addition of iERT to TAU will be examined at 12 months after baseline assessment, and at follow-up 24 and 36 months after baseline assessment. Additional clinical outcome assessments will take place 8 weeks, 14 weeks, and 6 months after start of treatment. Apart from a diagnostic interview (Mini International Neuropsychiatric Interview; MINI) [50] that will be conducted at baseline and at 12 months follow-up, all assessments consist of self-report measures that will be completed over the Internet. The Medical Ethical Committee of the VU University Medical Center has approved the study protocol. The study is registered at the Netherlands Trial Register, part of the Dutch Cochrane Center (NTR5822).

### Participants

Our target population consists of 200 adult outpatients with both a depression and a recent history of victimization. Inclusion criteria are: (1) a diagnosis of a major depressive disorder according to DSM-IV criteria, with or without a concurrent anxiety disorder other than Obsessive-Compulsive Disorder; (2) an indication for evidence-based psychotherapy for MDD or anxiety disorder according to clinical practice guidelines; (3) having been victim of at least one violent crime (physical assault, sexual assault, or threat) over the past three years; (4) access to a computer or tablet with Internet connection; and (5) an age of 18 years or older. Exclusion criteria are: (1) insufficient understanding of the spoken and written Dutch language; (2) psychotic symptoms; (3) bipolar disorder; (4) concurrent substance dependency that requires intervention; and (5) current high risk for suicide that requires intervention.

### Sample size

Our primary outcome variable is the number of incidents of victimization (count data); therefore, a Poisson distribution is assumed. A priori sample size calculation was performed under this assumption, using the asypow package for R 3.0. Since we are the first to examine effects of an intervention aimed at reducing revictimization in depressed patients, it is difficult to determine an exact estimate of the effect size for the main outcome measure. We expect that patients in the experimental condition will have experienced 30% less incidents at the endpoint in comparison with patients in the control condition, which we consider a realistic and clinically meaningful effect.

A previous study showed that 34% of depressed patients had been victim of at least one violent crime during one year [17]. On average, these patients had been victim of 3.30 (SD = 4.32) violent crimes in a one-year period – after two outliers (2%) who reported an extremely high number of incidents were deleted. Based on these results, we calculated that the expected average number of incidents over a period of 12 months after baseline, in a subsample of recently victimized patients, will be 3.0. Since the observed variance in the previous study indicated an overdispersed Poisson distribution, we performed numerical simulation in R for our sample size calculation, with adjustment for the expected overdispersion. Based on this simulation, we will need a minimum of 95 patients in each condition to demonstrate a difference between conditions of 30% in total incidents with α = .05, two-sided, and 1-β = .80. To account for missing information due to patient dropout, we aim to include 200 patients in total.

### Procedure

#### Recruitment and consent

Participants will be recruited at the mood and anxiety disorder departments of GGZ inGeest and Arkin: the two largest mental health institutes in Amsterdam, the Netherlands. All patients referred to one of the participating sites will be screened for eligibility by a clinician during regular intakes, and all eligible patients will receive written study information directly after intake. Patients who agree to be approached by a researcher will be contacted by telephone after one week by a research assistant, who will provide further information about participation in the study and will make an appointment for the screening interview. The screening will preferably take place within one to maximum four weeks before the patient starts with TAU, and will be performed either by telephone or face-to-face – depending on the patient’s preference.

**Fig. 1:**
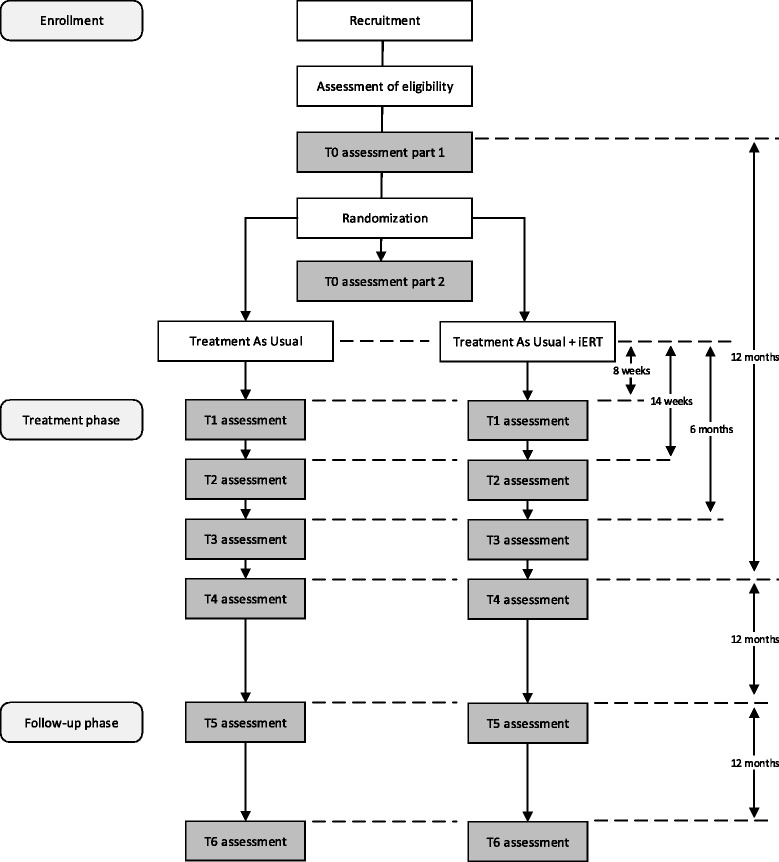
Trial flow chart

At the screening, inclusion and exclusion criteria will be assessed in detail by a research assistant. First, the MINI International Neuropsychiatric Interview (MINI 5.0) [50] will be administered to assess a current diagnosis of depression and other Axis I disorders. In addition, three slightly adapted questions of the Safety Monitor addressing the experience of three types of violent crime (physical assault, sexual assault, or threat) will be asked. A definition and examples of each type of crime will be provided. If the patient reports having experienced at least one violent crime over the past three years, he or she will be asked to briefly describe the crime to ensure that he or she was violently victimized. All patients who meet the criteria will sign an informed consent prior to the first assessment, and will be included in the study. To decrease the burden for participants, the baseline assessment will be divided into two parts that both consist of online administered self-report questionnaires. The first part is to be completed within one week after the screening, and the second part is to be completed preferably within a week after the first part. Figure 1 provides an overview of the trial design.

#### Randomization and procedure

Randomization will be carried out after completion of the first part of the baseline assessment. Randomization of participants in every participating site will be performed by a research associate of the data management department of GGZ inGeest, who is not familiar with the subject, nor involved in providing any kind of mental health care. Randomization will take place at an individual level, stratified by mental health care site, using a computer-generated block randomization schedule. To ensure that an equal number of patients will be allocated to TAU and TAU + iERT, the allocation ratio will be 1:1. To prevent selection bias, researchers and outcome assessors will be blind to block size and order, and will not have access to the randomization schedule. Due to the nature of treatments, blinding of participants and therapists to treatment condition is not feasible. Blinding research assistants to treatment allocation is not applicable to most outcome measures, since these will be administered online. Research assistants responsible for administering the MINI interview by telephone at 12 months after baseline will be blinded to treatment allocation. To promote data quality, all research assistants will be trained in administering the MINI interview. Audio recordings will be made of every MINI interview, which will be used in supervision sessions. Since all other measures concern web-based questionnaires that are filled out by the participants themselves, double data entry procedures are not applicable.

During the treatment phase, assessments will be administered at 8 weeks, 14 weeks, and 6 months after start of TAU in both the experimental and the control group. Follow-up assessments will be administered at 12, 24, and 36 months after the first baseline assessment. Except for the MINI, which will be assessed in a telephone interview at 12 months after baseline, all assessments will be conducted online. Patients receive an e-mail with a link to the self-report questionnaires, and those who have not completed an assessment within 4 days will receive a reminder via e-mail. Patients who have not completed the assessments after one week will be approached via telephone by a research assistant. If necessary, online assessments can also be completed over the telephone by an assistant who is blinded to treatment condition. Participants will be compensated with a voucher of 15 euros per assessment for both the first and second part of T0, T1, T2, T3, T5 and T6, and a voucher of 30 euros for T4.

Additional questionnaires will be sent out in the TAU + iERT condition: a visual analogue mood scale (VAMS) is to be completed online after every iERT-session in the online treatment platform, and the System Usability Scale (SUS) and Technical Alliance Inventory (TAI) are to be filled out after completion of iERT. The SUS will also be sent out once to each iERT-therapist. Table 1 provides an overview of all instruments per assessment.

**Table 1:** Overview of instruments per assessment

Instrument	T0_1_	T0_2_	T1	T2	T3	T4	T5	T6^a^
Mini International Neuropsychiatric Interview	x					x		
Safety Monitor	x					x	x	x
Inventory of Depressive Symptomatology	x		x	x	x	x	x	x
Difficulties in Emotion Regulation Scale	x		x	x	x	x	x	x
Visual Analogue Mood Scale	x		x^b^	x	x	x	x	x
Positive and Negative Affect Schedule	x		x	x	x	x		
Demographic questionnaire	x							
Posttraumatic Diagnostic Scale		x			x			
Childhood Trauma Questionnaire	x							
List of Threatening Experiences		x						
Brief Symptom Inventory		x			x			
Utrechts Coping List		x				x		
Ruminative Response Scale		x	x	x	x	x		
Pearlin Mastery Scale		x	x	x	x	x		
Dysfunctional Attitude Scale		x	x	x	x	x		
Self-Esteem Rating Scale	x							
Inventory of Interpersonal Problems		x				x		
NEO Five Factor Inventory			x					
EuroQol 5D-5 L	x				x	x		
Trimbos questionnaire on Costs associated with Psychiatric Illness	x				x	x		
Working Alliance Inventory				x				
Client Satisfaction Questionnaire				x				
Technical Alliance Inventory				x^c^				
System Usability Scale				x^c^				

^a^*T0*_*1*_*:* baseline part 1, *T0*_*2*_*:* baseline part 2, *T1:* 8 weeks after start of TAU, *T2:* 14 weeks after start of TAU, *T3:* 6 months after start of TAU, *T4:* 12 months after baseline, *T5:* 24 months after baseline, *T6:* 36 months after baseline

^b^Will also be assessed after each iERT-session in the experimental group

^c^Will only be assessed in the experimental group

### Interventions

#### Internet-based emotion regulation training (iERT)

The experimental intervention, iERT, is an abbreviated and slightly adapted online version of the Affect Regulation Training (ART) [44]. ART is a transdiagnostic structured group intervention that aims to enhance emotion regulation skills and consists of techniques from dialectical behavioral therapy, Systems Training for Emotion Predictability and Problem Solving program (STEPPS), emotion-focused therapy, Cognitive Behavioral Therapy (CBT), mindfulness-based interventions, self-compassion trainings, and problem-solving therapies. Although ART uses some techniques that are also included in CBT, ART explicitly and exclusively focuses on enhancing emotion regulation skills as opposed to CBT, which mainly targets cognitive and behavioral antecedents of depression [51]. In ART, the patient acquires seven emotion regulation skills, which the patient learns to use one-by-one by means of psycho-education and exercises. Subsequently, the patient practices each newly acquired skill in combination with previous skills using an audio file that guides him or her through the cycle of acquired skills [51].

To develop an online version of ART for patients with a major depressive disorder in specialized mental health care institutes in the Netherlands, ART was first translated into the Dutch language by an educated medical translator. Subsequently, the material was abbreviated and simplified, since all depression experts whom we consulted considered the original version too complicated and extensive for online use in moderately to severely depressed patients. iERT therefore focuses on a selection of four instead of seven skills: (1) non-judgmental awareness of emotions (*Awareness*); (2) acceptance and tolerance of emotions (*Acceptance*); (3) analysis of emotions (*Analysis*); and (4) active modification of emotions (*Modulation*). This selection was based on a literature review that we conducted, which indicated these emotion regulation skills to be highly relevant for both depressed patients [33, 35] and victimized patients [32, 52]. Lastly, in consultation with the developers of ART, we translated the Dutch, abbreviated protocol into an online version that will be provided on a secured online platform.

iERT consists of 6 sessions of approximately 30 min. After each session, a trained psychologist will provide feedback and guidance using secured e-mail within the online platform. In iERT, general psycho-education about the four skills and the origin, functions, and characteristics of emotions is offered in videos. These videos take 3 to 8 min to watch, and are designed to provide information in a clear, comprehensible, and visually attractive manner. Each session starts with one or two short psycho-education videos, followed by an exercise. Each session ends with an audio file exercise that combines the newly acquired skill with the previous skills. The participant is stimulated to practice with the audio file exercise at least once per week. When participants have mastered the skills by means of in-session theory, examples and exercises, their iERT-therapist stimulates them to start practicing their skills in daily life. The first iERT-session will be introduced in the experimental condition after the fourth session of TAU; from that moment on, the iERT-sessions will run parallel to TAU. A new session will become available weekly, provided that the participant has completed the previous session. The therapists will monitor whether their patients have worked through the previous session in time, and will contact them within two weeks if they have not.

#### Treatment as usual (TAU)

TAU is defined as the routine care that participants receive when treated for a depression or anxiety disorder in outpatient mental healthcare. The type of treatment may vary, and may include all evidence-based types of psychotherapy that are part of the treatment guidelines for depression and anxiety disorders – combined with pharmacotherapy, if necessary. TAU will mainly consist of Cognitive Behavioral Therapy (CBT) [53] or Interpersonal Psychotherapy (IPT) [54], often combined with antidepressant medication. TAU will be offered at each participating site, and will not be interfered with during the study in both the experimental group and the control group. TAU may be offered individually or in a group, and face-to-face, online, or blended. We will closely monitor the type and amount of treatment through patient records, the patient’s self-reported health care utilization, and a therapist questionnaire.

#### iERT-therapists

iERT will be provided by therapists of the e-mental health clinic of GGZ inGeest, who have ample experience with delivering internet-based treatment for depression. All therapists will receive an extensive training on how to deliver iERT – including how to use the online intervention and how to provide written feedback within the theoretical framework of ART. In order to ensure treatment fidelity, a detailed treatment manual with standardized written feedback templates will be available to guide therapists through the iERT-treatment. In addition, iERT-therapists will regularly receive supervision. A random sample of each therapist’s written feedback will be analyzed and discussed in supervision sessions. For all patients, the iERT-therapist will be a different therapist than their TAU-therapist. Prior to iERT, the TAU-therapist provides necessary information regarding the patient’s diagnosis and treatment progress to the iERT-therapist – a procedure for which the patient has provided written consent.

### Primary outcome measure

#### Violent victimization

Violent victimization will be measured with section 4 of the Safety Monitor (Dutch version: Veiligheidsmonitor) [55], which is developed by the Dutch Ministry of Security and Justice. The Safety Monitor strongly resembles the International Crime Victimization Survey (ICVS) [56] and is used by Statistics Netherlands (CBS) to measure victimization annually on a large scale. The Safety Monitor is an adequate self-report instrument that assesses victimization of 11 different crimes, subdivided into three categories: violent crimes, property crimes, and vandalism. In this study, we will only assess the violent crimes category, which consists of three subcategories: physical assault, sexual assault, and threat. Physical assault will be defined as deliberately hurting a person physically, with or without the use of a weapon. Sexual assault will be defined as unwanted sexual touching. Lastly, threat will be defined as threatening to hurt a person physically or sexually, without using actual violence. For each of these crimes, participants are asked whether they have become victim of that crime in the past 5 years. If so, participants are asked whether they have experienced that crime in the past 12 months, and, when answering affirmative, how frequently they experienced that crime in the past 12 months. The primary outcome measure is the total number of violent victimization incidents at 12 months after baseline (T4).

### Key secondary outcome measures

#### Diagnosis of depression

The presence of a current and lifetime diagnosis of depression in the past and present will be assessed with section A and B of the MINI International Neuropsychiatric Interview (MINI; version 5.0) [50]. The MINI is a structured, clinician-administered diagnostic interview that is widely used to assess the presence of psychiatric disorders based on the Diagnostic and Statistical Manual of Mental Disorders (Fourth edition; DSM-IV) and the International Classification of Diseases (Tenth revision; ICD-10). The MINI demonstrated a good Kappa score of 0.84 in diagnosing MDD, with a sensitivity of 0.96 and a specificity of 0.88 in comparison with the patient-rated version of the Structured Clinical Interview for DSM-IV (SCID) [57]. The validated Dutch version of the MINI [58] will be used.

At T0, sections A and B of the MINI will be conducted to assess current and lifetime depression. In addition, sections C, D, I, J, K, and L of the MINI will be administered to assess the presence of suicidality, bipolar disorders, posttraumatic stress disorder, alcohol abuse or dependency, drug abuse or dependency, and psychotic disorders, respectively. At T4, section A will be administered again in a telephone interview, to determine remission rates of depression. In addition, the sections covering suicidality, posttraumatic stress disorder, and alcohol and drug abuse or dependency will be administered again.

#### Depressive symptom severity

Depressive symptoms will be assessed with the 30-item Inventory of Depressive Symptomatology – Self Report (IDS-SR; Dutch translation) [59, 60], which is a self-report questionnaire designed to measure depressive symptom severity. The IDS-SR includes all diagnostic DSM-IV criteria for major depressive disorder (MDD), as well as melancholic, atypical, and commonly associated symptoms for MDD (e.g., anxious mood, irritable mood). The IDS-SR covers five core symptom domains: vegetative symptoms, cognitive changes, mood disturbance, endogenous symptoms, and anxiety symptoms. All items are rated on a 4-point Likert scale from 0 to 3, and are equally weighted in the total score. The IDS-SR has highly acceptable psychometric properties and has been demonstrated to be sensitive to treatment effects in depressed outpatients [60–62]. The IDS-SR will also be used to determine response and remission rates. Response rates will be defined as a reduction in baseline total score of the IDS-SR of at least 50%. Remission rates will be defined as the absence of a diagnosis of depression according to the MINI and an IDS-SR score of < 18 [60].

### Other secondary outcome measures

Other secondary outcome measures are:

- Quality of life, as measured with the EuroQol 5D (EQ-5D-5 L) [63];
- Healthcare costs and productivity losses/gains, as measured with the Trimbos/iMTA questionnaire
- on Costs associated with Psychiatric illness (TiC-P) [64];
- Psychopathology, as measured with the Brief Symptom Inventory (BSI) [65];
- PTSD symptomatology, as measured with the Posttraumatic Diagnostic Scale (PDS) [66];
- Interpersonal functioning, as measured with the Inventory of Interpersonal Problems (IIP-C) [67];
- Coping style, as measured with the subscales *Active tackling* and *Avoidance* of the Utrecht Coping List (UCL) [68].

### Process variables

#### Emotion dysregulation

Emotion dysregulation will be measured with the Difficulties in Emotion Regulation Scale (DERS) [69]. The DERS is a 36-item self-report scale that assesses clinically relevant emotion regulation difficulties across various dimensions, represented in six subscales: non-acceptance of emotional responses, difficulty engaging in goal-directed behavior, impulse control difficulties, lack of emotional awareness, limited access to emotion regulation strategies, and lack of emotional clarity. The DERS has demonstrated high internal consistency, good test-retest reliability [69–71], and adequate construct and predictive validity [69].

#### Positive and negative affect

The 20-item Positive and Negative Affect Schedule (PANAS) [72] will be used to assess positive and negative affect at the time of assessment; both represented by 10 items that are rated on a 5-point Likert scale. Each item consists of an affective state, such as “excited”, “proud”, and “attentive” for the positive affect subscale, and “distressed”, “ashamed”, and “guilty” for the negative affect subscale. The PANAS has good internal consistency and validity [72]. The Dutch version [73] yields acceptable to good psychometric properties as well, with a Cronbach’s alpha of .77 and .87 for the positive and negative affect subscales, respectively [74].

#### Sad mood

Sad mood will be measured by a digital version of the 1-item Visual Analogue Mood Scale (VAMS), which consists of a line that runs from zero to 100, with the descriptors “happy” located on the left side and “sad” on the right side. A higher score represents a sadder mood. Patients are asked to rate their current mood by placing a cursor on the line, with the following instruction: “You can answer the following question by replacing a cursor on the line from 0 to 100. At this moment, I feel…”. The VAMS has been used previously in studies examining the influence of sad mood on relapse and recurrence of depression [75, 76]. In the experimental condition, the VAMS will be assessed after each iERT-session as well.

#### Brooding

The 5-item Brooding subscale of the Ruminative Response Scale (RRS; Dutch version) [77, 78] assesses the dysfunctional thinking pattern of drawing one’s attention to problems and their consequences. For each item, respondents are asked to rate the extent to which it reflects their responses to sadness on a 5-point Likert scale. An exemplary item is “I think: why do I always react this way?”. The Brooding subscale has satisfactory internal consistency (Cronbach’s alpha = .77) [77] and predicted depression both cross-sectionally [79] and prospectively [77].

#### Dysfunctional attitudes

The Dysfunctional Attitude Scale form A (DAS-A) is a self-report scale designed to measure patterns of negative thinking in depressed patients [80], with good internal consistency and validity [81–83]. In this study, the shorter 17-item version will be used (DAS-A-17). Respondents are asked to what extent they agree with each of 17 dysfunctional assumptions on a 7-point Likert scale. A confirmatory factor analysis demonstrated the Dutch version of the DAS-A-17 to yield good psychometric properties in terms of reliability and convergent construct validity [84]. 

#### Sense of control

Sense of control is measured with the 7-item Mastery scale [85]. Mastery concerns the extent to which one perceives oneself to be in control of events and factors that influence one’s life [85]. Respondents are asked to rate the extent to which they agree with seven statements on a 5-point Likert scale, for example: “There is really no way that I can solve some of the problems that I have.” The Mastery scale has adequate psychometric properties [85, 86].

### Potential moderators

- Demographic characteristics, collected at baseline;
- Childhood trauma, as measured with the Childhood Trauma Questionnaire (CTQ) [87];
- Alcohol and drug abuse or dependency, as measured with the MINI 5.0 [50];
- Self-esteem, as measured with the Self-Esteem Rating Scale Short-Form (SERS-SF) [88];
- Personality dimensions, as measured with the Neuroticism-Extraversion-Openness Five Factor Inventory (NEO-FFI) [89];
- Negative life events, as measured with the List of Threatening Experiences [90];
- Working alliance, as measured with the Working Alliance Inventory – Short Form (WAI-SF) [91].

### Other variables of interest

#### Treatment evaluation

The 8-item Client Satisfaction Questionnaire (CSQ-8) [92] will be administered to assess patient satisfaction with treatment in both the experimental group and the control group. The CSQ has high internal consistency (α = .93) [93].

In addition, the 10-item System Usability Scale (SUS) [94] will be administered to participants receiving TAU + iERT, after they have finished iERT-treatment regularly or prematurely. The SUS assesses participants’ subjective perception of the usability of a technology system. The questionnaire will be administered once to each iERT-therapist as well. The SUS has been demonstrated to be reliable and robust [95].

#### Contextual information of victimization

The Safety Monitor assesses contextual information about the most recent incident of each violent crime experienced in the past 12 months: where the incident took place, whether the participant knew the perpetrator and what their relationship was, and whether the participant reported the crime to the police. To obtain more detailed information on the most recent victimization incident, we extended the Safety Monitor with supplemental questions, such as: “did you use substances or alcohol prior to the incident?”.

#### Victimization of non-violent crimes

Apart from violent crimes, we will ask participants whether and how frequently they have experienced the following non-violent crimes in the past 12 months: burglary, car theft or theft of another motor vehicle, pickpocketing or non-violent robbery, theft of other property, fraud, and vandalism.

#### Perpetration

Perpetration will be measured with the extended version of the Safety Monitor. For each violent crime, participants will be asked whether they have ever committed that crime. If so, participants are asked whether and how frequently they have permitted that crime in the past 12 months. For each crime they committed in the past 12 months, they will be asked whether they simultaneously were both victim and perpetrator, and if so, whether they committed the crime in self-defense. Prior to these questions, participants are reminded about the confidentiality of the assessment.

#### Safety perception and perceived controllability of victimization

Section 3 of the Safety Monitor assesses safety perception by asking participants whether and how often they feel unsafe. We extended this section with four questions regarding safety perception and perceived controllability of victimization, such as: “To what extent do you consider yourself able to prevent ending up in hazardous situations?” and “How likely do you think it is that you will fall victim to each of the following during the next twelve months?”.

### Data analysis

#### Effectiveness

Primary data analyses will be conducted in conformity with the intention-to-treat paradigm. In addition, per-protocol analyses will be performed. Missing data will be addressed using multiple imputation. Treatment effect regarding the primary outcome measure will be modeled with Generalized Linear Mixed Models (GLMM) with a Poisson distribution, since the primary outcome measure consists of count data. Treatment effect concerning the secondary outcome variables will be modeled using GLMM as well, taking into account distributional characteristics of the data. Considering the expected skewness of the data, we assume that removal of outliers will be necessary.

To assess the magnitude of treatment effects on primary and secondary outcome measures, Cohen’s d between groups effect sizes for each time point will be calculated. Effect sizes of d = .9 are considered large, effect sizes of d = .45 are considered moderate and effect sizes of d = .15 are considered small [96]. Furthermore, multilevel mediation models will be used to examine various potential treatment mediators. Although the major question of this study concerns victimization, we will determine differences between both conditions regarding response and remission rates of depression as well.

#### Cost-effectiveness

The economic evaluation will be conducted alongside the randomized controlled trial and will be performed according to the intention-to-treat principle. With regard to the economic evaluation, we will take into account the CHEERS statement [97] and the 2015 ISPOR good research practices task force report on cost-effectiveness analysis alongside clinical trials [98]. Using a societal perspective, we will evaluate the relationship between costs – direct medical costs, participant costs and productivity losses, as measured with the TiC-P – and health outcomes of TAU + iERT and TAU alone at 12 months after baseline.

We will take into account four types of costs: (1) the costs of offering the intervention (TAU + iERT or TAU only); (2) costs stemming from general health care uptake besides TAU + iERT or TAU only, including the costs of medication; (3) patients’ out-of-pocket expenses (e.g. travel costs, leisure time spent on receiving care); and (4) costs stemming from productivity losses due to absenteeism or reduced efficiency while at work (presenteeism). Health care costs will be valued based on standard cost prices reported in the Dutch guideline for economic evaluation [99]. Costs due to productivity losses will be based on the gender- and age-specific labor costs. Data on resource use (health care uptake) and productivity losses will be collected with the widely-used TiC-P [64]. Using this approach, cumulative costs over the full trial period will be obtained from the cost estimates at times of the data collection waves.

We will perform both a cost-effectiveness analysis with incidents of victimization as effect measure and a cost-utility analysis using QALYs. QALYs will be based on preferences from general population samples to derive value sets to calculate the EQ-5D-5 L health utilities for the Netherlands. Using the area under the curve (AUC) method, the periods between the measurement waves will be multiplied by the utility of the health state in that period. This allows for the computation of quality adjusted life years (QALYs) over the entire trial period.

Incremental cost-effectiveness ratios (ICERs) and cost-effectiveness acceptability curves (CEAC’s) will be calculated. To estimate the uncertainty around the ICERs, 5000 bootstrapped samples will be extracted and plotted on cost-effectiveness planes. These data will also be used to plot Cost Effectiveness Acceptability Curves (CEACs). One-way sensitivity analyses and/or scenario analyses directed at assessing the impact of uncertainty in the main cost drivers will be performed to gauge the robustness of our findings. In addition, a sensitivity analysis in which covariate-adjusted CEACs can be constructed will be conducted using net benefit regression methods [100, 101].

### Data management

Data management of this research project will be performed by the Data Management department of GGZ inGeest, which is not otherwise involved in the project. The project data will be securely saved on the central server of GGZ inGeest. The IT department professionally maintains the server and daily performs a backup of all data. All data will be pseudonymized using unique study codes that will be used to code and file all electronic information. Only designated members of the research team will have access to a secured file with the key that links this code to the participant’s identity. Since all outcome measures concern either web-based questionnaires that are filled out by the participants themselves or a computer-based interview that is filled out digitally by a research assistant, hard copy storage of questionnaires is not applicable. All informed consents will be stored both electronically and in hard copy, with the hard copies stored in a locked cabinet at each participating site.

## Discussion

This paper describes the study protocol of a randomized controlled trial aimed at assessing the effectiveness of the addition of iERT to TAU in reducing violent revictimization, depressive symptoms, and emotion regulation difficulties in previously victimized, depressed patients. Despite the high prevalence rates and societal burden of victimization in psychiatric patients, there remains a paucity of knowledge of risk factors and predictors regarding violent victimization in depressed patients. Accordingly, evidence-based interventions aimed at reducing victimization and revictimization are still scarce. To the best of our knowledge, this study is the first to examine the effectiveness of an intervention aimed at reducing revictimization in depressed patients. We will investigate the hypotheses that the addition of iERT to TAU is effective and cost-effective in decreasing incidents of revictimization in depressed patients compared to TAU alone. Furthermore, this study will examine whether the addition of iERT to TAU leads to a significant improvement on secondary outcome measures. If iERT is effective in reducing incidents of revictimization, it can be implemented in mental health care.

Major strengths of the current study are the long follow-up period of 36 months and the large amount of validated, clinically relevant outcome measures that are administered at multiple assessments during the treatment phase, which enables us to gain insight into the underlying mechanisms of revictimization. The most important concern of this study will be treatment adherence, since internet-based treatments are associated with high dropout levels [102, 103]. Weekly therapist guidance and the relatively small amount of iERT-sessions is expected to enhance treatment adherence, but it will remain an important challenge of this trial. To prevent study dropout, participants will receive monetary compensation for all assessments.

In conclusion, the prospective and longitudinal design of this study provides a unique opportunity to thoroughly examine revictimization rates and a variety of relevant secondary outcomes, mediators, and moderators over a period of three years.

## Abbreviations

ART: Affect Regulation Training
AUC: Area Under the Curve
BSI: Brief Symptom Inventory
CBT: Cognitive Behavioral Therapy
CEAC: Cost-Effectiveness Acceptability Curves
CHEERS: Consolidated Health Economic Evaluation Reporting Standards
CSQ-8: 8-item Client Satisfaction Questionnaire
CTQ: Childhood Trauma Questionnaire
DAS-A: Dysfunctional Attitude Scale form A
DERS: Difficulties in Emotion Regulation Scale
EQ-5D-5 L: EuroQol 5D-5 L
ERT: Emotion Regulation Training
GLMM: General Linear Mixed Models
ICER: Incremental Cost-Effectiveness Ratio
ICVS: International Crime Victims Survey
IDS-SR: Inventory of Depressive Symptomatology – Self Report
iERT: Internet-based Emotion Regulation Training
IIP-C: Inventory of Interpersonal Problems
ISPOR: International Society for Pharmaco-economics and Outcomes Research
MINI: Mini International Neuropsychiatric Interview
NEO-FFI: NEO Five Factor Inventory
PANAS: Positive and Negative Affect Schedule
PDS: Posttraumatic Diagnostic Scale
PTSD: Post-Traumatic Stress Disorder
QALY: Quality Adjusted Life Years
RCT: Randomized Controlled Trial
RRS: Ruminative Response Scale
SERS-SF: Self-Esteem Rating Scale Short-Form
SMI: Severe Mental Illness
STEPPS: Systems Training for Emotional Predictability and Problem Solving
SUS: System Usability Scale
TAI: Technical Alliance Inventory
TAU: Treatment As Usual
TiC-P: Trimbos/iMTA questionnaire on Costs associated with Psychiatric illness
UCL: Utrecht Coping List
VAMS: Visual Analogue Mood Scale
WAI-SF: Working Alliance Inventory – Short Form
